# Advanced and Careful Hospital Nutrition Reduces Respiratory Assistance and Hospitalization in Patients With COVID‐19: A Retrospective Longitudinal Observational Study

**DOI:** 10.1002/hsr2.71440

**Published:** 2025-11-02

**Authors:** María Jesús Vega‐Bello, Sandra Carrera‐Juliá, Xandra Luque, Ignacio Turró Bautista, Mari Ángeles Navarro, Teresa Pérez, María Eugenia Dulcich, Concepción Manrique, Mari Luz Moreno

**Affiliations:** ^1^ Department of Physiology Universidad Católica de Valencia San Vicente Mártir Valencia Spain; ^2^ Department of Nutrition and Dietetics Universidad Católica de Valencia San Vicente Mártir Valencia Spain; ^3^ Gastronomy and Nutrition Area Clínica Universidad de Navarra Madrid Spain; ^4^ Instituto de Investigación Sanitaria La Fe Hospital Universitari i Politècnic La Fe Avenida Fernando Abril Martorell Valencia Spain; ^5^ Department of Basic Sciences Universidad Católica de Valencia San Vicente Mártir Valencia Spain

**Keywords:** COVID‐19, hospital nutrition, hospital stay, respiratory assistance

## Abstract

**Background and Aims:**

Hospital nutrition is an essential component of the multidisciplinary care of COVID‐19 patients. Malnourished COVID‐19 patients present more symptoms, complications, and require longer hospitalization times. Therefore, the aim of the present investigation was to study the influence of an advanced and careful hospital nutrition on COVID‐19 patients' evolution and treatment.

**Methods:**

One hundred and twenty‐two patients suffering from COVID‐19 and hospitalized in the “Clínica Universidad de Navarra” (CUN) (Madrid) and attended by professionals from the hospital's gastronomy and nutrition area participated in the study. Hospital meals were subjected to advanced culinary techniques such as sous vide cooking, special electrical oven, food marination, and steam and pressure cooking. Clinical, food intake, respiratory assistance, and hospital stay were assessed.

**Results:**

COVID‐19 patients present comorbidities and digestive symptoms which impair food intake and adherence to a healthy diet. The use of advanced and careful hospital nutrition using special culinary techniques that maintain the organoleptic characteristics of the meals improves patients' intake, reducing the requirement for respiratory assistance and shortening hospital stay.

**Conclusion:**

Advanced hospital nutrition is key in managing patients affected by COVID‐19 because it influences their progression and treatment.

## 1 Introduction

COVID‐19 is a severe acute respiratory syndrome caused by the coronavirus 2 (SARS‐CoV‐2). In 2020, this condition was recognized by the WHO as a pandemic with deadly consequences [1]. The disease leads to a secondary inflammatory syndrome [2] that is associated with hypermetabolism and increased patients' energy requirements [3]. It can result in poor nutrition intake because COVID‐19 is associated with a wide variety of symptoms that reduce appetite and compromise normal food intake, such as nausea, vomiting, diarrhea, fever, cough, myalgia, fatigue [4], and alterations in taste and smell [5]. The most frequently observed clinical comorbidities in patients with COVID‐19 are diabetes, renal insufficiency, cardiovascular disease, dementia, and chronic obstructive pulmonary disease (COPD), which contribute to a worse nutritional status and prognosis [6].

Recent research data indicate that nutrition is a key and influential factor in the treatment and recovery of the patient. Thus, the nutritional status of the patient is a key factor in COVID‐19, while the disease itself is an influential factor for nutrition deterioration [7]. Specifically, malnourished patients present more symptoms, complications, and require longer hospitalization times [8]. This is noteworthy because malnutrition has a high incidence among hospitalized patients with COVID‐19 [9].

Therefore, the aim of the present investigation was to study the influence of excellent hospital nutrition on COVID‐19 patients and treatment.

## 2 Materials and Methods

### 2.1 Type of Study and Participants

A retrospective longitudinal observational study was conducted. The study sample consisted of those patients who came to the hospital emergency room of “Clínica Universitaria de Navarra” (CUN) (Madrid) from June 2020 to September 2023 with COVID and, due to the severity of their symptoms, were hospitalized. The inclusion criteria were men and women over 18 years old and admitted to the CUN with a positive diagnosis of COVID‐19 who were able to eat orally. The exclusion criteria were pregnant or lactating women, patients with alcohol and other drug abuse, patients that used enteral or parenteral artificial nutrition, and those who refused to participate in the study. During the 3 years of the study, 71 patients refused to participate in it. Finally, a sample of 122 patients was obtained.

### 2.2 Ethical Concerns

The study was conducted according to the guidelines of the Declaration of Helsinki [10] and approved by the Ethics Committee of Clínica Universidad de Navarra (Navarra, Spain) (procedure number: 2020.060). Written informed consent was obtained from all subjects involved in the study.

### 2.3 Procedures

All procedures were carried out at the clinical facilities of the CUN. The procedures were carried out as part of the routine periodic revision of patients by members of the gastronomy and nutrition area of the CUN.

### 2.4 Clinical Aspects

During the admission and follow‐up of each patient, a brief clinical history was registered by members of the gastronomy and nutrition area of the CUN and the attending physicians. Additionally, the medical notes were consulted using the CUN's database. With all this information, a general characterization of the study sample was obtained regarding the patients' age, hospital stay, and current or previous tobacco consumption. The patient's clinical comorbidities were also recorded. Digestive and intestinal symptoms and meal intake (good intake, good tolerance, good appetite, increased intake) were monitored during the patient's periodic visits by healthcare professionals. *Good intake* was considered when the patient ate 70% of the meal; *good tolerance* when the patients did not experience any intestinal discomfort after eating the meal; *good appetite* when the patient had the desire of eating after visualizing the meal; *increase intake* when the patient moved progressively from 70% to 100% of the meal intake.

### 2.5 Anthropometric Evaluation

An anthropometric assessment was conducted, recording the patient's body weight and height at the beginning of the hospital stay. Both measurements were taken following the guidelines of the International Society for the Advancement of Kinanthropometry (ISAK) by an ISAK Level I anthropometrist using specialized anthropometric equipment.

For body weight measurement, a portable clinical scale model SECA (Hamburg, Germany) with a maximum capacity of 150−200 kg and an accuracy of 100 g was used. The patient stood on the center of the scale with arms resting at the sides of the body. For patients unable to stand, a chair scale SECA 952 (Hamburg, Germany) with a maximum capacity of 200 kg was used for weighing while seated.

Height measurement was obtained through two methods. One method involved consulting available data in the medical records of each patient. In other cases, a SECA (Hamburg, Germany) 220 model stadiometer with 0.1 cm precision was used.

Body mass index (BMI) was calculated and categorized as follows: normal weight (≥ 18.5–24.99 kg/m²), underweight (≤ 18.5 kg/m²), grade I overweight (25–26.9 kg/m²), grade II overweight (27–29.9 kg/m²), or obesity (≥ 30 kg/m²) [11].

### 2.6 Food Technology

The objective of the gastronomy and nutrition area at CUN is to improve hospital nutrition by integrating diet as a useful part of treatment to reduce malnutrition and hospital stay. To achieve this, efforts are made to improve patient intake, adherence to prescribed diets, increase appetite, and ensure good tolerance.

For this purpose, the facilities of the CUN's kitchen were used, where a series of additional culinary techniques were applied, different from those used in the preparation of a standard hospital diet in Spain.

The technique of sous vide cooking was employed, involving cooking food in vacuum‐sealed bags at controlled temperatures and times. This technique improves the texture and flavor of the food while maintaining good levels of antioxidant nutrients [12]. A vacuum packing machine SAMMIC Model SE420 (Guipuzkoa, Spain) was used for this purpose. It is particularly useful for cooking meat and fish as it allows the texture to remain juicy and tender [13], similar to the use of cooking by probe, which was also employed.

The use of frying was avoided by using the Rational SCC101 (Barcelona, Spain) electrical oven, which allows maintaining a crispy texture while reducing the production of acrylamides secondary to the Maillard reaction, which have potential carcinogenic properties [14].

To reduce the use of salt, food marination was employed, which ensured pleasant and adequate flavor without the need to increase sodium consumption, particularly useful for patients with hypertension or cardiovascular risk.

For vegetables, steam and pressure cooking were used to maintain texture and provide vitamins and minerals, as demonstrated in a previous study conducted on broccoli [15]. In this case, Moduline CVE031 (Veneto, Italy) steam pressure oven was used.

For sweetening, a slow and continuous treatment of fruit was used instead of adding sugar. This way, syrups, compotes, jams, and fruit smoothies were prepared without any added sugar and with a lower amount of sucrose.

### 2.7 Statistical Analysis

Statistical analysis was carried out using Python software (version 3.9) with the SciPy 1.11 library. The correlation between categorical variables was assessed using the chi‐square test, following the recommendations of Agresti for categorical data analysis [16]. Pre‐specified analyses included comparisons of hospital stay duration, respiratory support requirements, and food intake using ANOVA. Exploratory analyses included subgroup comparisons for digestive symptoms and comorbidities, assessed through post hoc pairwise tests. Assumptions of homoscedasticity were checked using Levene's test, and normality was tested with the Shapiro−Wilk test [17]. In cases where normality was not met, the Kruskal−Wallis test was applied [18]. Effects were considered significant when *p* < 0.05.

## 3 Results

### 3.1 General Characteristics of the Patients

The analysis of the general characteristics of the study sample (Table 1) revealed that it included 122 patients: 65 males and 57 females.

**Table 1:** General characteristics of the study sample.

	Men	Women
Total (*n* = 122)	65	57
Age (years)*	66.37 ± 15.52	71.18 ± 12.41
Age (years) minimum–maximum	41−94	41−95
Weight (kg)*	84.91 ± 15.26	68.15 ± 10.43
BMI (kg/m^2^)	26.74 ± 4.50	25.64 ± 3.36
Smoker* (currently or previously)	30	13
Hospital stay (days)*	13.01 ± 9.26	10.28 ± 9.58
Hospital stay (days) minimum–maximum	4−54	5−27

*Note:* The descriptive analysis values are shown as sample number, mean ± SD, and minimum–maximum interval. All aspects are considered significant (*) with a value of *p* < 0.05.

Abbreviation: BMI, body mass index.

The mean age for men was 66.37 ± 15.52 years, and for women, the mean age was 71.18 ± 12.41 years, which was significantly higher (*p* = 0.034).

The average weight for men (84.91 ± 15.26 kg) was significantly higher (*p* < 0.05) than the average weight for women, which was 68.15 ± 10.43 kg.

In men, the mean BMI was 26.74 ± 4.50 kg/m^2^. For women, it was 25.64 ± 3.36 kg/m^2^. In both sexes, patients were in a situation equivalent to grade I overweight.

Regarding tobacco consumption, a total of 30 men were identified with current or past smoking habits, which was significantly higher (*p* < 0.05) than the number of women (13) with the same habit. Specifically, five men were active smokers at the time of hospital admission, and 25 were ex‐smokers. Among women, a total of 13 had a history of tobacco use, with four being active smokers at the time of admission and nine being ex‐smokers.

As for hospital stay, men had an average stay of 13.01 ± 9.26 days, which was significantly longer than women (10.28 ± 9.58 days).

### 3.2 Clinical Comorbidities

The analysis of clinical comorbidities (Table 2) revealed that 22.35% of patients suffered from hypertension, with 13.64% being men and 8.71% being women.

**Table 2:** Clinical comorbidities of the patients.

Disorder or syndrome	Total (%)	Men (%)	Women (%)
Hypertension	22.35	13.64	8.71
Diabetes mellitus	4.55	3.79	0.76
Hypercholesterolemia	2.27	0.76	1.52
Dyslipidemia	11.74	6.44	5.30
Hyperuricemia	1.14	1.14	0.00
Hiatal hernia	2.65	0.76	1.89
Chronic gastritis	0.76	0.76	0.00
Diverticulosis	1.89	1.89	0.00
Diverticulitis	1.14	0.00	1.14
Cholecystectomy	1.52	1.14	0.38
Hepatic steatosis	1.89	1.89	0.00
Hypothyroidism	4.55	0.38	4.17
Acute renal failure	1.14	0.76	0.38
Chronic renal failure	1.14	0.76	0.38
Chronic obstructive pulmonary disease (COPD)	4.55	2.27	2.27
Pulmonary emphysema	1.14	0.76	0.38
Obstructive sleep apnea syndrome	2.27	1.52	0.76
Alzheimer's disease	1.52	1.14	0.38
Parkinson's disease	1.52	1.14	0.38
Benign prostatic hyperplasia	3.03	3.03	0.00
Prostate cancer	1.89	1.89	0.00
Depressive syndrome	4.55	1.52	3.03

*Note:* The frequency of clinical comorbidities in the study sample is presented as a percentage (%). The percentage of affected patients is shown in total, as well as divided into men and women.

Diabetes mellitus affected 4.55% of the patients, predominantly affecting men (3.79%). However, hypercholesterolemia (2.27%) predominated in women (1.52%) compared to men (0.76%). Dyslipidemia was present in 11.74% of patients, with 6.44% being men and 5.3% being women. However, no significant differences were found between the two sexes.

Hyperuricemia affected 1.14% of patients, all of whom were male. Hiatal hernia was determined in 2.65% of patients, with 1.89% being women. Chronic gastritis was not significant as it did not reach 1% of affected individuals in the study sample.

Diverticulosis occurred in 1.89% of patients, all of whom were men. Particularly, episodes of diverticulitis were only observed in women, at 1.14%. In the case of cholecystectomy, it was observed in 1.52% of patients, predominantly in men (1.14%). Hypothyroidism was determined in 4.55%, with up to 4.17% of those affected being women.

Both acute and chronic renal failure were determined in 1.14% of the sample, and both occurred in the same proportion by sex. COPD was determined in 4.55% of patients, with equivalent values in both sexes. Pulmonary emphysema was observed in 1.14% of patients. Obstructive sleep apnea syndrome affected 2.27% of patients, with 1.52% of those affected being men.

Regarding neurodegenerative diseases, Alzheimer's disease was present in 1.52%, as was Parkinson's disease. Both conditions had the same distribution based on the patient's sex and predominated in men (1.14%) compared to women (0.38%).

Regarding prostate disorders, benign prostatic hyperplasia occurred in 3.03% of men, while prostate cancer was identified in 1.89%. In reference to mood disorders, depressive syndrome affected 4.55% of patients, with 3.03% being women and 1.52% being men.

### 3.3 Digestive Symptoms

After analyzing the digestive symptoms during hospital stay (Table 3), a total of 19 patients were determined to suffer from nausea, with 14 of them being women, representing 74% of patients with this symptom. It is worth noting that this value was significantly higher (*p* < 0.05) compared to men, where five patients were identified, representing 26% of those affected. In the case of vomiting, a total of seven patients were identified, two men (29%) and five women (71%).

**Table 3:** Main digestive symptoms of the patients.

Symptomatology	Men *N* (%)	Women *N* (%)
Nausea*	5 (26)	14 (74)
Vomiting	2 (29)	5 (71)
Ageusia	27 (47)	31 (53)
Anosmia	29 (48)	32 (52)
Diarrhea	25 (45)	30 (55)
Constipation	15 (60)	10 (40)
Use of laxatives*	19 (73)	7 (27)

*Note:* Differences between men and women are considered significant (*) with a value of *p* ≤ 0.05.

Abbreviations: *N*, number of patients; %, percentage of affected patients.

In reference to chemosensory dysfunctions, a total of 58 patients suffered from ageusia and 61 from anosmia with no differences between sexes.

The number of patients with diarrhea (55) was higher in women (30, representing 55%) compared to men (25, representing 45%). Conversely, constipation was identified in a total of 25 patients and was higher in men compared to women. Specifically, 15 men (60%) compared to 10 women (40%). Particularly, the assessment of laxative use in a total of 26 patients was significantly higher (*p* < 0.05) in men (19, representing 73%) compared to women (7, representing 27%).

### 3.4 Relationship Between Hospital Nutrition, Respiratory Treatment, and Hospital Stay

Table 4 shows that those patients who had good intake and/or good tolerance and/or good appetite when admitted increased their intake during their hospital stay.

**Table 4:** Relationship between intake characteristics and respiratory assistance.

Variable	Variable	*p* value	Effect size
Good intake (*N* = 26)	Increase in intake (*N* = 25)	*p* < 0.001**	Phi (0.40)
Good tolerance (*N* = 19)	Increase in intake (*N* = 25)	*p* < 0.023*	Phi (0.27)
Good appetite (*N* = 16)	Increase in intake (*N* = 25)	*p* < 0.012*	Phi (0.29)
Good intake (*N* = 26)	Nasal glasses not required (*N* = 28)	*p* < 0.032*	Phi (0.23)

*Note:* All aspects are considered significant (*) with a value of *p* < 0.05 and (**) with a value of *p* < 0.001. Good intake was considered when the patient ate 70% of the meal; good tolerance when the patients did not experience any intestinal discomfort after eating the meal; good appetite when the patient had the desire of eating after visualizing the meal; increase intake when the patient moved progressively from 70% to 100% of the meal intake.

Abbreviation: *N*, number of patients.

The strongest correlation between variables was observed between “good intake” and “increase in intake” (*p* < 0.001). Additionally, a slight negative correlation was observed between “good intake” and “nasal glasses not required,” which is equivalent to saying that a patient who had good intake is less likely to require nasal glasses.

After observing that patients with good intake did not require the use of nasal glasses, we determined whether this group stayed fewer days in the hospital and therefore recovered earlier from COVID‐19 infection. It was found that patients who had good intake and did not use respiratory assistance (nasal glasses) stayed an average of 10 days in the hospital (95% CI: 8.76, 11.89), compared to the rest of the patients who remained hospitalized for an average of 15 days (Table 5) (95% CI: 12.01, 18.75), showing a statistically significant difference (*p* = 0.008, Cohen's *d* = 0.72).

**Table 5:** Hospital stay of patients according to the intake and use of nasal glasses.

	Hospital stay (days)	Confidence interval
Good intake and no nasal glasses requirement	10	[8.76, 11.89]
The rest of the patients	15**	[12.01, 18.75]

*Note:* Differences between patients with good intake‐no nasal glasses requirement and the rest of the patients are considered significant (**) with a value of *p* < 0.01.

## 4 Discussion

### 4.1 Clinical Characteristics of the Patients

In this study, it was determined that, in general terms, the mean age of patients affected by COVID‐19 corresponded to an advanced age. Generally, it is known that older individuals have a higher risk of getting infected by the COVID‐19 virus, which appears to be more lethal [19].

In relation to BMI, our results show that both men and women presented grade I overweight. This situation is considered a precursor to the development of obesity, which has a strong negative prognostic risk factor in COVID‐19 and can increase hospital mortality, requirement for hospitalization, and the use of mechanical ventilation [20]. Thus, a study reported that around 90% of COVID‐19 patients treated in intensive care units (ICUs) had a BMI greater than 35 kg/m² and required ventilatory support [21]. Moreover, many of these overweight patients were or had been smokers. In this line, most evidence has shown a strong relationship between smoking and COVID‐19 infection, due to the detrimental influence of tobacco on the respiratory system. Tobacco could influence virus transmission, and a long‐term smoker could increase the risk of developing the disease [22]. For this reason, the number of smoking patients was determined, and it was observed that the number of male smokers was significantly higher than that of females. Additionally, 4.55% of the patients presented COPD, a respiratory disease whose main cause is tobacco [23]. It is known that 50% of COPD exacerbations are caused by viral respiratory infections, which could suggest that SARS‐CoV‐2 would be an etiological factor in COPD patients, increasing the risk of mortality and the need for respiratory assistance [24].

The COVID‐19 patient is characterized by presenting associated clinical comorbidities that worsen the prognosis and evolution [25] and can affect more than 70% of hospitalized adult patients [26]. The most reported comorbidities are hypertension, diabetes mellitus, and cardiovascular disease [27]. In our study, we identified that 22.35% of patients presented hypertension and 11.74% presented dyslipidemia. Both factors contribute to a higher risk of cardiovascular disease. 4.55% of patients presented depressive syndrome, higher in women (3.03%) compared to men (1.52%). This result is in line with previous studies indicating that epidemiological data showed that women seem to be more likely to develop depression [28]. Regardless of the patient's sex, depression would promote the release of pro‐inflammatory cytokines in subjects with COVID‐19 [29], complicating the progression of the disease.

Symptomatic infection by SARS‐CoV‐2 also presents gastrointestinal involvement [30]. Our results confirm the presence of nausea, vomiting, ageusia, anosmia, diarrhea, constipation, and the need for laxatives. This clinical profile affected more than 50% of the patients. Gastrointestinal symptoms negatively influence the patient's food intake, leading to nutritional deficits or difficulties in nutrient absorption, resulting in a higher risk of malnutrition [31]. It is worth noting that there was a significantly higher need for laxative use secondary to constipation in men compared to women, despite constipation is more characteristic in women due to physiological conditions [32]. Therefore, our study indicates that SARS‐CoV‐2 could affect men more negatively in this aspect.

In patients with COVID‐19, the presence of overweight, clinical comorbidities, and digestive symptoms would impair the caloric and nutritional requirements. This highlights the relevance of providing an adequate hospital diet to ensure proper intake that leads to a good nutritional status and a better prognosis [33].

### 4.2 Relevance of Hospital Nutrition

Hospital nutrition is an essential component of the multidisciplinary care of COVID‐19 patients. Caloric and nutritional deficiency predisposes to a higher risk of malnutrition, which negatively affects immune system functionality [34]. In addition, malnutrition also affects hospitalization time. In a study conducted on 4311 COVID‐19 patients, it was concluded that malnutrition increases both hospitalization time and morbidity [35]. Several scientific studies have evaluated the role of hospital diet and nutritional interventions during the course of infectious diseases, particularly in contexts such as COVID‐19, pneumonia, and critical illness. Current evidence suggests that adequate hospital nutrition not only helps preserve patients' nutritional status but may also be associated with improved clinical outcomes. In hospitalized COVID‐19 patients, multiple studies have shown that dietary interventions can prevent nutritional decline and modulate the inflammatory response. For instance, a prospective pilot study using an immunonutrition formula enriched with protein and anti‐inflammatory compounds demonstrated significant reductions in inflammatory markers as well as a lower risk of malnutrition progression [36]. Similarly, an observational study in London across two COVID‐19 waves found that dietitian‐led interventions effectively halted weight loss among critically ill patients, highlighting the importance of individualized nutritional management [37].

Beyond COVID‐19, other infections also demonstrate the value of structured dietary support. A randomized trial in elderly patients hospitalized with pneumonia showed that a targeted nutritional intervention improved Mini Nutritional Assessment (MNA‐SF) scores and significantly reduced 6‐month readmission rates [38].

Training hospital staff in nutritional care has also been shown to improve nutritional status at both admission and discharge, while reducing hospital length of stay. A quasi‐experimental study involving over 580 patients demonstrated that such training nearly doubled the likelihood of maintaining adequate nutritional status and shortened hospitalization by almost 3 days [39].

In addition, hospital admission for COVID‐19 has a negative impact on the patient's emotional state, which can result in reduced appetite, leading to poor intake [40]. This is relevant because, as previously indicated, up to 4.55% of the study sample presented depressive syndrome.

COVID‐19 infection leads to chemosensory dysfunctions such as anosmia and ageusia [41]. Although alterations in smell and taste are not critical for health nor suppose a life‐threatening risk to the patient, the disruption in perceiving the sensory qualities of food is highly associated with the pleasure of eating. In COVID‐19, the presence of these alterations could lead to a loss of motivation for food intake [42]. This, coupled with a decrease or absence of appetite, could increase weight loss and malnutrition [43]. For this reason, when chemosensory dysfunction is identified, it is necessary to focus attention on the color and texture of hospital meals to maintain appetite with the aim of sustaining and/or increasing food intake [42].

Our research determined that patients who had good intake and/or good tolerance and/or good appetite upon admission increased their intake during their hospital stay, indicating that the carefully designed and prepared diet at the CUN was highly accepted and therefore successful. It was also identified that patients who had a good intake had a lower requirement for nasal glasses, demonstrating the significant impact of good hospital nutrition in treating COVID‐19 patients. This finding is relevant because maintaining good intake leads to less compromise of nutritional status, greater effectiveness of the immune response, and lower severity of respiratory symptoms [44]. In fact, respiratory difficulties limit the ability to intake solid and liquid foods due to coughing, dry mouth, or swallowing difficulties [45]. Additionally, patients who did not require respiratory support (nasal glasses) stayed a shorter period in the hospital.

All these positive results could be related to the use of advanced culinary techniques in the hospital nutrition service of the CUN. These culinary techniques ensure suitable organoleptic characteristics that enhance the palatability of the meals. Consequently, adherence to the hospital diet is improved, which may have contributed to increased intake in patients. Additionally, the use of these culinary techniques facilitates meeting the caloric and nutritional requirements of the patient. This, coupled with adequate intake, would result in a lower risk of malnutrition and better patient recovery, as it is known that at least 60% of patients with COVID‐19 experience malnutrition [46].

Therefore, our results highlight the importance of considering an early, adequate, and adapted nutritional support for patients with COVID‐19.

### 4.3 Limitations of the Study

In the assessment of the patient's nutritional status, it would be interesting to analyze more relevant anthropometric variables related to a state of malnutrition, such as: body perimeters, skin folds, and body composition (fat mass, muscle mass, and hydration status). This would allow a deeper analysis of the body composition of each patient that would help to understand the evolution of the nutritional status after the administration of the hospital diet. Similarly, biochemical analysis of serological values for proteins, vitamins, and minerals would provide valuable insights into the impact of hospital nutrition on COVID‐19 patients. In addition, a detailed assessment of the organoleptic characteristics of the menu (taste, smell, color, and texture) by the patient could be considered to further investigate the effectiveness of the culinary techniques. Finally, due to the lack of similar studies in Spain, it has not been possible to make a direct comparison with previous research, which highlights the importance of further studies in this line of investigation.

## 5 Conclusion

The implementation of an advanced and carefully designed hospital nutrition program plays a crucial role in the progression and treatment of patients hospitalized with COVID‐19. The use of specific culinary techniques that improve the organoleptic qualities of meals contributed to better intake, increased tolerance, and improved appetite among patients. These nutritional improvements were significantly associated with a lower need for respiratory assistance and a shorter length of hospital stay. Given the high prevalence of malnutrition, comorbidities, and digestive symptoms among COVID‐19 patients, the integration of high‐quality nutrition into hospital care emerges as an essential therapeutic strategy. Therefore, early and personalized nutritional support should be prioritized as a standard component in the multidisciplinary management of COVID‐19 and potentially other acute illnesses.

## Author Contributions

**María Jesús Vega‐Bello:** conceptualization, data curation, writing – original draft. **Sandra Carrera‐Juliá:** formal analysis, writing – original draft. **Xandra Luque:** data curation. **Ignacio Turró Bautista:** formal analysis. **Mari Ángeles Navarro:** formal analysis. **Teresa Pérez:** data curation. **María Eugenia Dulcich:** data curation. **Concepción Manrique:** conceptualization, supervision. **Mari Luz Moreno:** conceptualization, supervision, writing – review and editing. All authors have read and approved the final version of the manuscript.

## Conflicts of Interest

The authors declare no conflicts of interest.

## Transparency Statement

The lead author, Mari Luz Moreno, affirms that this manuscript is an honest, accurate, and transparent account of the study being reported; that no important aspects of the study have been omitted; and that any discrepancies from the study as planned (and, if relevant, registered) have been explained.

## Data Availability

Data sharing is not applicable to this article as no data sets were generated or analyzed during the current study. The corresponding author, Mari Luz Moreno, had full access to all of the data in this study and takes complete responsibility for the integrity of the data and the accuracy of the data analysis.
